# Impacting Career Choices of Historically Underserved Secondary Students by Designing Near-Peer Directed Acid–Base Thematic Laboratory Activities to Enhance STEM Interest

**DOI:** 10.1021/acs.jchemed.3c00434

**Published:** 2023-08-21

**Authors:** Abha Verma, Mehnaaz F. Ali

**Affiliations:** Department of Chemistry, Xavier University of Louisiana, New Orleans, Louisiana 70125, United States

**Keywords:** High School Education, STEM Education, Near-Peer Mentoring, Laboratory Enrichment, Mobile Outreach, Inquiry-Based Discovery Learning

## Abstract

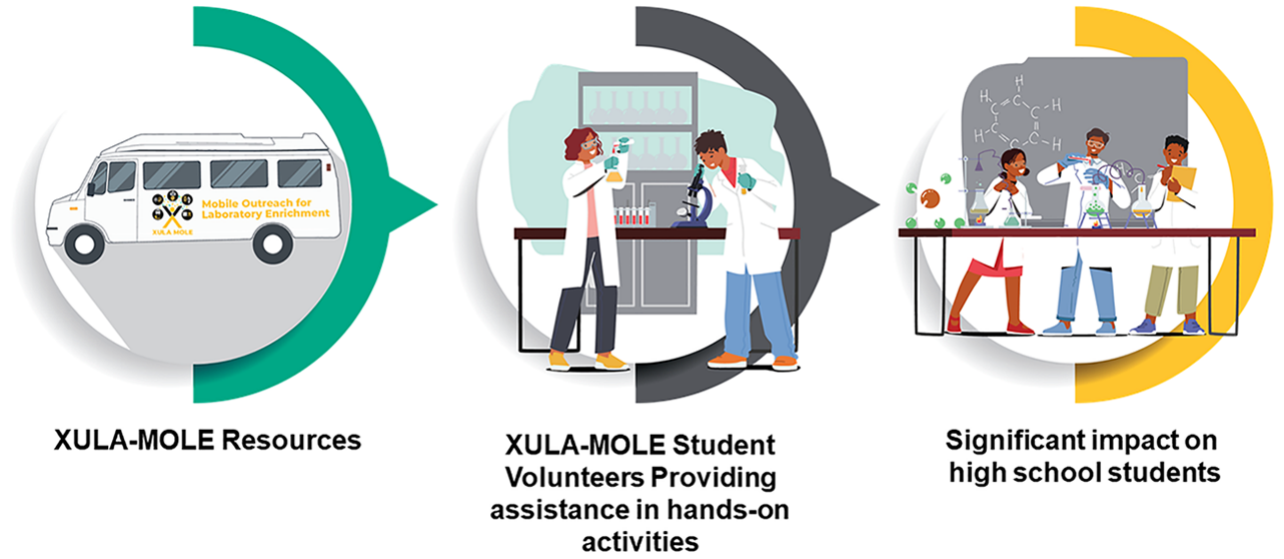

The current study describes preliminary findings from the Xavier University of Louisiana Mobile Outreach for Laboratory Enrichment (XULA-MOLE) project, which is a collaboration between Xavier University of Louisiana (XULA), a Historically Black and Catholic University, and participating 9th–12th grade classrooms in the central New Orleans area with a historically underserved student population. The project described here is geared toward providing laboratory enrichment to enhance student learning and impact student career interest in STEM fields, especially in classrooms with a much-needed “hands-on” laboratory experience which is unavailable due to a lack of resources. In this case study, we will present and discuss the inquiry-based laboratory modules for the topic area of acids and bases. These modules were created with careful thought and revision by XULA undergraduate STEM students. The experimental modules were based on the curriculum that participating teachers were discussing in the high-school classroom during the semester. The active-learning efforts were carried out during 6 weeks of the semester to provide a sustained and impactful resource for the participating classrooms. Since both groups of students (XULA-MOLE students and the high school students) were from underrepresented groups there was a strong sense of shared interest and dynamic near-peer mentorship. The project outcomes were measured using both formative and summative assessments indicative of preliminary successes in impacting career interests and increasing the content knowledge of participating high-school students.

## Introduction

### The Need

The U.S. Bureau of Labor Statistics stated in 2022 that several of the predicted fastest-growing jobs in the future will require STEM training^1^ with a projected 1,064,000 new STEM jobs in 2031. However, according to the National Assessment of Educational Progress, only 22% of students in high schools are at or above proficiency in STEM subjects.^2^ Particularly underserved high schools are more affected since they lack resources, which unequivocally widens the achievement gap since these students are not only unprepared to work in STEM laboratories but also have had little opportunity to cultivate an interest in science-related fields.^3,4^ This problem has grown exponentially in the state of Louisiana, especially as an aftermath of the damages caused by Hurricane Katrina in 2005 and Hurricane Ida in 2021.^5^ The state has been ranked one of the worst in the context of college readiness, high school graduation, and national assessments.^6^ Despite the rebuilding efforts after the storm devastation, ACT math and science scores from 2015 to 2019 indicate a steady decrease as seen in Table 1.^7^ Analysis of state education reform by the nonprofit organization *New Schools for New Orleans* revealed gaps in the existing curriculum with deficiencies existing within the math and science curricula resulting in a misalignment with the State’s college academic standards.^8^

**Table 1:** ACT Scores Comparison between Louisiana (LA) and the National Averages for the Past Few Years^a^

ACT Scores^b^							
District/Region	2014	2015	2016	2017	2018	2019	2022
Louisiana	19.2	19.4	19.5	19.6	19.3	18.9	18.1
Nation	21.0	21.0	20.8	21.0	20.8	20.7	19.8
LAACT Math scores	N/A	18.9	18.8	18.8	18.5	18.2	17.4
LAACT Science scores	N/A	19.4	19.6	19.6	19.1	18.8	18.3

a Source: ACT data file; Louisiana Department of Education.^7^

b Due to the onset of the COVID-19 pandemic, data for 2020–21 were unavailable.

**Figure 1:**
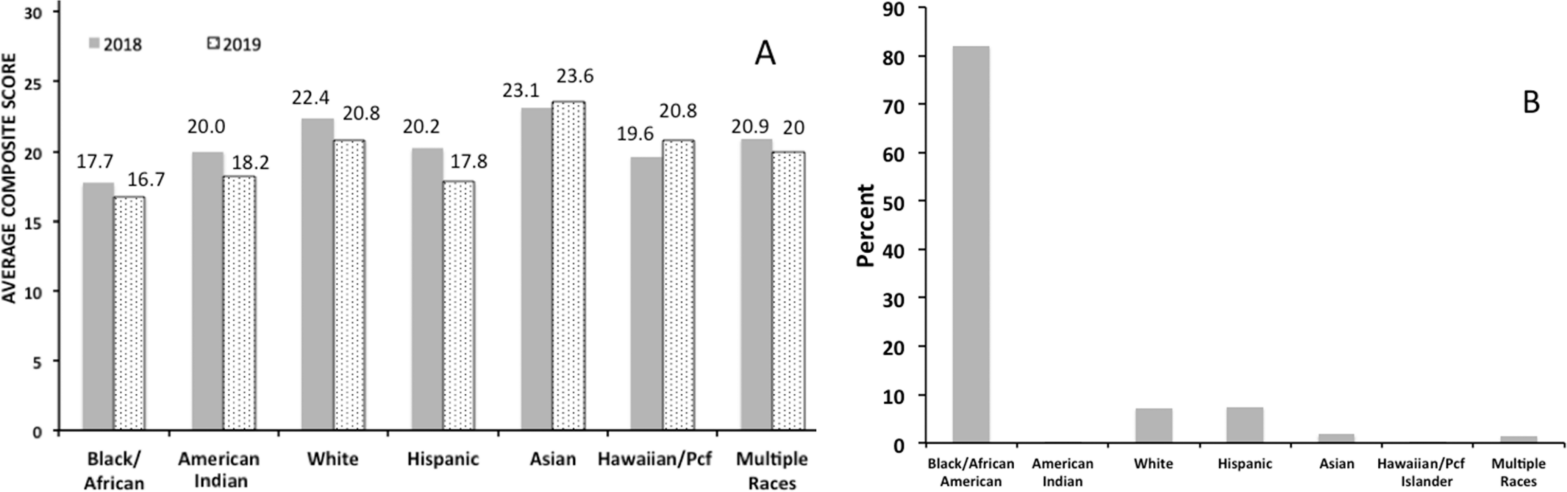
(A) Comparison of 2018 and 2019 Louisiana Composite ACT scores by race. (B) Race/ethnicity group breakdown for New Orleans secondary school students (Source: Louisiana Believes).^9^

The deficiencies of the misaligned curricula have had an even more drastic impact on historically underserved students in the state as seen in Figure 1. Not only have the assessment scores for Black/African American, American Indian, and Hispanic student groups dropped from 2018 to 2019, but also the Black/African American cohort of students is scoring over two points below the state average in 2019. The continuing shortages in effective available curricula are especially significant since our target area of New Orleans has over 80% Black/African American students who are directly impacted by these education reforms.^9^

### Our Intent

A review of the literature indicates several community colleges and universities have stepped up to bridge the gap by offering access to authentic laboratory interaction and experiences with professionals in STEM fields.^10^ Some of these programs are targeting high school students from underrepresented populations and have highlighted the key components making those programs effective.^11,12^ The Xavier University of Louisiana’s Mobile Outreach for Laboratory Enrichment (XULA-MOLE) project is one of these efforts and was launched to address the scarcity of laboratory experiences for high school students, especially from historically underserved institutions of secondary education in the Greater New Orleans areas.

Xavier University of Louisiana (XULA) is a Historically Black and Catholic University, nationally recognized for its science, technology, engineering, and mathematics (STEM) curriculum, while remaining close to its liberal arts roots. XULA’s mission is to create a more just and humane society by preparing its students to assume roles of leadership and service in a global society. This preparation takes place in a diverse learning and teaching environment that incorporates all relevant educational means, including research and community service. Aligned to this mission, the XULA-MOLE project was initiated with the intent of supporting and enriching STEM education at high schools with historically underserved students to stimulate their interest in pursuing a career in STEM and related fields and thus reduce the gap of inequity of access to high-quality science education.

### Our Aim

The primary objective of this project was to provide middle and high school students with four to five hands-on science experiments each semester while enhancing XULA undergraduate students’ understanding of science content by eliciting their help in developing these science experiments. The specific high school outcomes were:

i. Producing hands-on activities that correlated with classroom material.
ii. An increased positive view of science.
iii. An increased interest in a career in STEM fields shown by the participating students.

This project is unique from other research experiences for high school students in several aspects. Primarily, it is designed to develop near-peer mentorship since the undergraduate students from XULA play a predominant role in mentoring high school students, and second, the project utilizes a mobile format which allows us to reach secondary students over a wider area in Greater New Orleans, while tailoring the curriculum for each individual classroom. The undergraduate student volunteers, which make up the XULA MOLE project, play an important role as they are responsible for developing the experiments and modules, and this in turn helps them gain valuable experience in science communication and teaching. Approximately, 85% of students at XULA are reported as African-American, which leads to shared interests and personalized mentoring experiences for the historically underserved secondary students.^13^

A primary aim includes providing secondary school students with an opportunity to engage in scientific research and gain valuable hands-on experience in a laboratory setting within their own school environment. The XULA-MOLE project provides all of the necessary materials and reagents for the tailored experiments. To further lessen the burden on the school and the teachers, a group of XULA student volunteers along with at least one Team Lead (also a XULA undergraduate student) are also sent for the school visits to conduct the hands-on learning experiments and small-group instruction. Herein, we will describe a six-week hands-on laboratory experience for high school students from historically underserved populations in New Orleans during Fall 2022 with the intent of providing a resource for other groups and organizations also looking to address challenges with experiential learning resources within the secondary school system.

## Methods

### Project Design

This XULA-MOLE project designed for high school students (Grades 9th to 12th) and conducted during the Fall of 2022 was primarily based on the framework that effective science education is dependent on the student having opportunities to learn about both the *process* and the content of *science*.^14^ As indicated by several studies, inquiry-based laboratory experiments^15^ have the ability to address both criteria with the careful selection of appropriate research topics and activities. Our inquiry-based modules were designed to positively impact high school students’ interest in STEM projects and ultimately evolve into an increased career interest in a STEM field.

The high schools of Louisiana have a high turnover of science teachers in most of the underserved public schools.^16^ Moreover, the teachers are trained minimally to develop inquiry-based laboratory curricula that align with their classroom science lessons. Discussions with participating STEM teachers confirmed this challenge and highlighted the severe shortage of resources both in the form of time and materials provided to the teachers to carry out more than 1–2 experiments per semester at a maximum.

Our approach for project design was to:

i. Formulate two different testable guiding research questions stemming from topics being discussed within the high school classroom.
ii. Develop inquiry-based activities to serve as exploration guides for the high school students for each of the modules in a school year.
iii. Have the high school students conduct the inquiry-based exercises aligned with the curriculum and reflect on the activities.
iv. Provide prepared activities to teachers for use with future classes.

### Recruiting Schools for Participation

Our project was piloted in four schools in the Greater New Orleans area that had existing challenges with science and math metrics and teacher turnaround. The participating science teachers’ schedules for the semester were obtained to determine viable time slots for classroom visits for a six-month period. The available time slots were cross-referenced with the availability of the XULA students, and a single visit time per week was selected for each classroom for the semester.

XULA students who volunteered to be part of the project and had matched with the available time slots traveled to these participating schools along with all laboratory equipment and reagents, where they delivered a short lecture on the topic to be covered that week regarding acid–bases and then guided/conducted experiments with the high school students.

### Recruiting XULA-MOLE Students

Recruitment flyers were created and posted around campus for all STEM field students, with the interest form presented as a QR code for easy access. Additionally, XULA’s Chemistry Club which is run and organized by XULA Chemistry major students was informed about this project via email and a recruitment visit to their orientation meeting. Different student-led University groups were also informed about the opportunity to work with the XULA-MOLE project as a mechanism to complete service obligations. Over 40 undergraduate students had applied, of which 15 students were finally selected (based on their match with the classroom timings) as XULA-MOLE volunteers for Fall 2022. The breakdown included 7% freshmen, 33% sophomores, 40% juniors, and 13% seniors. Most of the XULA student volunteers (95%) were female, and all of the students were underserved minority students.

Three XULA students (seniors) were also hired as XULA undergraduate student Team Leads (XUSTL), based on letters of reference and performance in STEM courses at XULA.

Based on the schedules cross-matched between the XULA-MOLE students including all of the XULA undergraduate student volunteers (XUSV) and XULA undergraduate student Team Leads (XUSTL), available classroom times, XULA-MOLE students were assigned schools for weekly visits where they led the classroom curriculum discussion in a small-group format. The XULA-MOLE students also helped design the inquiry-based modules for the chosen acid–base topic. These weekly modules were aligned with classroom instruction and the Next Generation Science Standards (NGSS).^17^ Additionally, the modules focused on the design and implementation of our guiding question (in this case, acids and bases-focused) by being broken up into smaller explorations that were more easily accessible for the high school students.

## Curriculum Development

As part of the design phase and prior to the dissemination of the lessons within classrooms, XULA-MOLE students were trained during *prep meetings* to be mindful of safety concerns, to give lectures, to distribute responsibilities among each other, and to guide the high school students through the hands-on experiment. The majority of the volunteers had the opportunity to deliver the lectures on separate visits, while others managed and oversaw the small groups during the allocated time slot. The curriculum development was initiated using the following steps:

### Safety Training

All of the XULA-MOLE students were required to participate in the Chemistry Department’s “safe laboratory practices” seminar and pass the safety quiz prior to attending the sessions. All Chemistry Department faculty and staff are required to undergo similar training annually.

On the first visit to the classrooms, the high school students were also given safety training by XULA-MOLE students, followed by completing the safety training questionnaire to ensure proper knowledge regarding the handling of chemicals and waste.

### Mentor Training for XULA Student Volunteers

An essential part of the XULA-MOLE Fall 2022 project focused on training the XULA undergraduate students on how to teach and why to teach rather than what to teach.^18^ Although the XULA students have typically progressed past the point of their high school mentees both in career progression and age, mentoring interactions can only be successful with a scaffolded approach to mentor training.^19^

Each week throughout the semester, the project faculty involved met with the XULA-MOLE students’ team for over a two hour block, to lead discussions on *communication*, *active listening techniques*, and *cultural competency*. Both the faculty and the students were trained via different programs for preparing mentors and advisors such as the NIH BUILD-funded Preparing Mentors and Advisors at Xavier (P-MAX) program. These meetings and different trainings commenced 2 weeks prior to the first visit to the high school classrooms.

Project faculty meetings with the XULA Undergraduate Student Team Leaders (XUSTL) were held separately for discussions, brainstorming ideas, and recruitment of the XULA Undergraduate Student Volunteers (XUSV), along with logistical planning of the project.

### Topic Selection for Fall 2022 Program

We aimed for the high school students to work on the experimental modules that would be based on topics that the teacher will be teaching during the semester of Fall 2022. The modules were small vignettes exploring different aspects of the main theme for the semester (in this case, acids and bases). For this curriculum-based outreach project, our approach was to plan the hands-on activities and experiments for students with a diverse readiness.^20,21^

The topic of acid and bases was selected after careful planning with the high school teachers of the participating classrooms. This topic was selected from the currently used high school Pearson Chemistry textbook^22^ which discusses the concept of *Stone Erosion of Famous Structures* around the globe as a prelude to introducing acids and bases. The laboratory experiment topic of acid–base was also aligned to the high school topics put forth by the NGSS which provided the framework for the Louisiana State standards for science.^23^

In order to better engage the participating New Orleans high school students, the concept of stone erosion was chosen to lead to an inquiry-based exploration^24^ on “What causes degradation of New Orleans cemeteries”. There are over 40 cemeteries scattered around the city and located in and around residential areas, and it is very likely that the high school students have seen them and can engage with the exploration.

### Program Activities and Resource Contents

**Figure 2:**
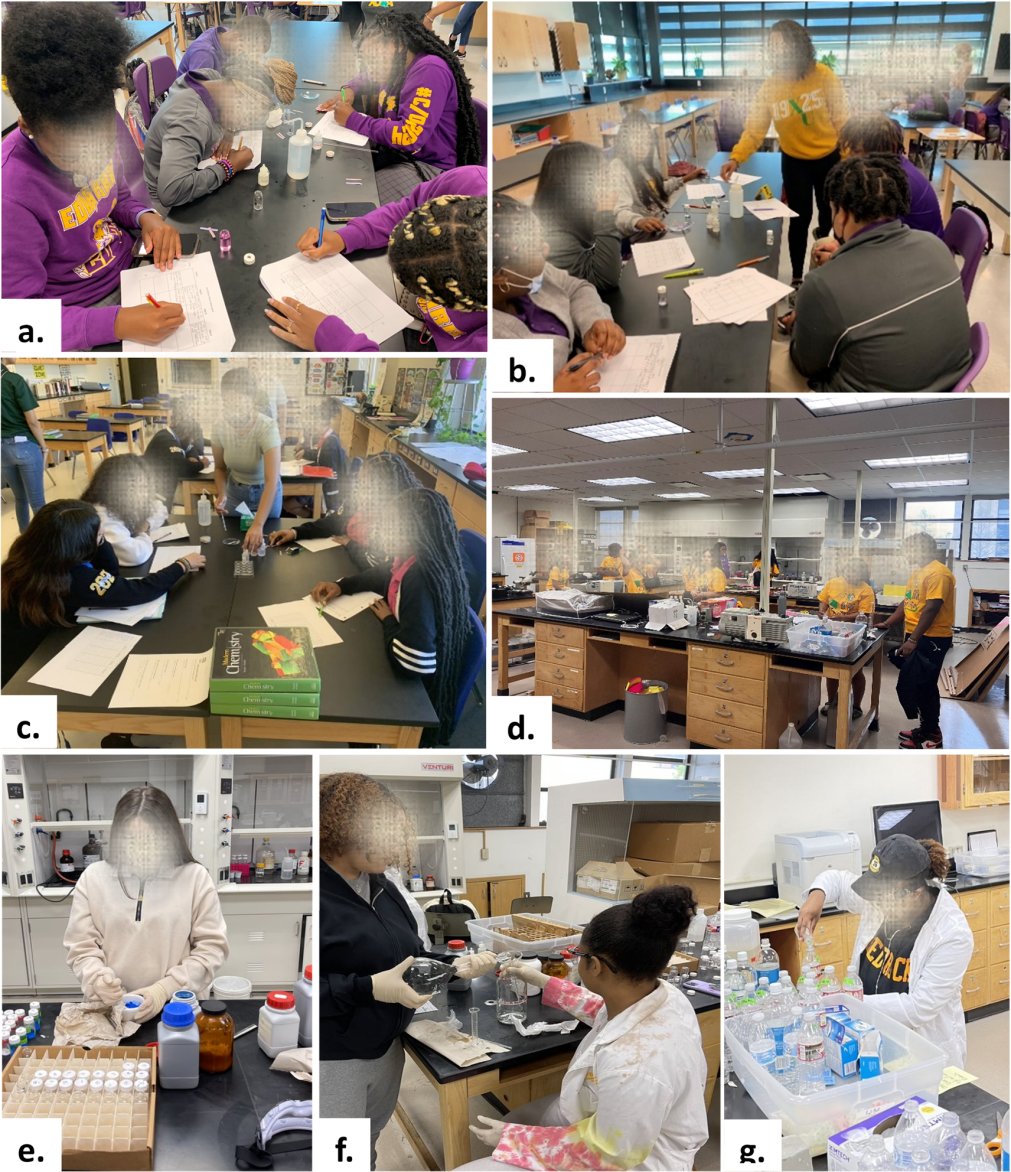
(a–c) High school students interacting with XULA students and performing the acid–base experiments. (d–g) XULA students preparing experiments and undergoing training. The faces of all students were concealed for privacy.

The XULA-MOLE program for Fall 2022 was initiated at four high schools, collaborating with high school teachers who were enthusiastic about the outcomes of using inquiry-based science experiments in their classrooms. The inquiry-based laboratory enrichment was carried out via six classroom visits during that semester in each of the four participating classrooms. Figure 2 depicts the interaction between the XULA-MOLE student teams and high school students. The discussion and accompanying experiments were designed to be more appropriately challenging for grades 11th–12th in comparison to activities for the 9th–10th grades. Examples of such differences included an inquiry that expands the understanding of the 3 models of acids and bases.^25^

We also wanted to incorporate the 5E^26^ instructional strategy—engagement, exploration, explanation, elaboration, and evaluation—in the teaching plan to increase students’ understanding, to facilitate inquiry as seen in Table 2.

**Table 2:** Teaching Plan for the Acid–Base Topic Designed for High School Students

Week	Plan for Training and Objectives	Teaching Activities
1.	Ice breaker Peer mentoring activity; Preassessment on interest in STEM activities and career. Assess preactivity content knowledge; Discuss guiding questions and engage students in constructing an investigative plan	**Engage:** Safe Laboratory practices presentation and discussion scientific process; assessment of baseline interest in STEM activities and career; Peer mentor, the XUSVs engaged the students in having a discussion on what is occurring with the cemeteries. High school students discussed prior knowledge of acid–bases in general and specifically about New Orleans cemeteries. Discussion of safety concerns related to acid–bases and in general.
2.	Students were introduced to the concept of acid–bases; Designing Concept map for Acids and Bases	**Explore:** Students worked in small collaborative groups to classify acids and bases. Used pH paper and other indicators as well as conductivity to classify substances.
3.	Test experiment (Match guiding question to experiments)	**Explore:** Students worked in small collaborative groups to explore different concentrations of acids and bases; neutralization of acids and bases and construct grade dependent model of acid and base.
4.	Complete background material. Research materials used to construct New Orleans cemeteries (Significance to the real world)	**Elaborate:** Design an experiment to test materials similar to the cemetery and write observations in a notebook. Discussions with XUSV peer mentors were also held regarding the scientific process. Students took a postactivity assessment.
5.	Peer-discussion with XULA students (Evaluations and Feedback)	**Evaluate:** Students were given an article on acid wear and asked to explain the process and provide suggestions for preventing tooth deterioration from acid. Students took a summative assessment by writing a CER with their collaborative groups on how to prevent tooth decay.
6.	Presentation of the project by students	**Explain:** Student groups presented posters with observations from the experiment and findings to the class as a summative assessment which was recorded for each group and assessed using nonproject faculty.

Week 1 involved the assigned XULA Undergraduate Student Team Leader discussing a safe laboratory practices presentation to the high school students and teachers. This first visit also assessed the high school students’ current career interests and gauged their interest in STEM activities. A short *prediscussion* assessment on their acid–base knowledge content was also administered during the first week. All of these sessions were facilitated completely by the XULA undergraduate student volunteers with help from the science teacher monitoring the classroom.

For week 2, students explored the neutralization of a weak acid with a weak base, indicated by the use of phenolphthalein and a conductivity meter. During week 3, students performed experiments that included the determination of the acidity/basicity of many solutions including multiple concentrations of different acids and bases such as NaOH and CH_3_COOH. In week 4, the concept of concentration was introduced by comparing the effect of different concentrations of nitric acid and sulfuric acid on various minerals such as calcium bicarbonate and granite, which were obtained as stone chips in alignment with the degradation of cemeteries. During week 5, which served as a summative evaluation, the pH of different drinks like Arizona Tea and Sprite were tested out and their effect on tooth deterioration was explored using concepts learned from the previous modules.

### Modules

The development of modules was crucial to this program, as they were used as a tool to guide the high school students through the laboratory experience and provided a scaffolded teaching approach to the XULA MOLE volunteer students. Developing modules with corresponding questionnaires and reflections also ensured that the high school students received structured instruction, which their teachers could reuse in future classroom discussions and for concept reinforcement.

Since modules were developed in concert with the science teachers, the high school students were able to relate what they were learning currently with the hands-on experiment, making the experience more meaningful and memorable. Datasheets were developed for each of the weeks for high school students to record their hypotheses, observations, and inferences from the experiments for the hands-on activity. Separate “lecture” and “materials needed” documents were also prepared as a guide for XULA-MOLE students and new classroom implementations. This strategy was also important to provide a consistent instructional plan for all of the participating classrooms. Week four’s Lecture and Datasheet are provided in the Supporting Information.

## Results

### Program Participation

The program participants included high school students who participated in the mobile laboratory enrichment activities, science teachers who invited us to their classrooms, and XULA students who served as near-peer mentors. Greater than 95% of the students were underrepresented minorities.

For Fall 2022, there were approximately 126 high school student participants in the mobile laboratory visits (treatment and comparison groups). We had four teachers who participated in the mobile laboratory sessions and 18 XULA undergraduate students that served as near-peer mentors.

### Program Surveys

The evaluation process and informed survey tools were approved by the Xavier University of Louisiana IRB (approval # 835). All assessments as well as surveys were kept anonymous and stored responsibly, as required by the IRB. The evaluation of this project includes data from both the high school and the undergraduate students. This information along with the verbal feedback from students and teachers helped assess the success of our curriculum objectives as well as the overall satisfaction with the project design.

The *“Is Science Me?”* survey was initially developed as an instrument to study an ethnically and economically diverse set of students, to explore why some students who were once interested in science, engineering, or medicine left the field while some students continued on in the STEM fields.^27^ Our participating high school students completed a modified version of this survey, so we could evaluate and gather input to better inform XULA-MOLE project design and effectiveness.

The survey was administered on the first day of classroom visits and on the last class day of the six-week period to determine the impact of the project activities on the interest of students pursuing science. All of our data were evaluated by an external evaluator.

The data was used to ascertain the:

i. Impact of the laboratory experiments on the high school students’ views on science and the work that scientists do.
ii. Interest of high school students in pursuing a career in STEM fields.

**Figure 3:**
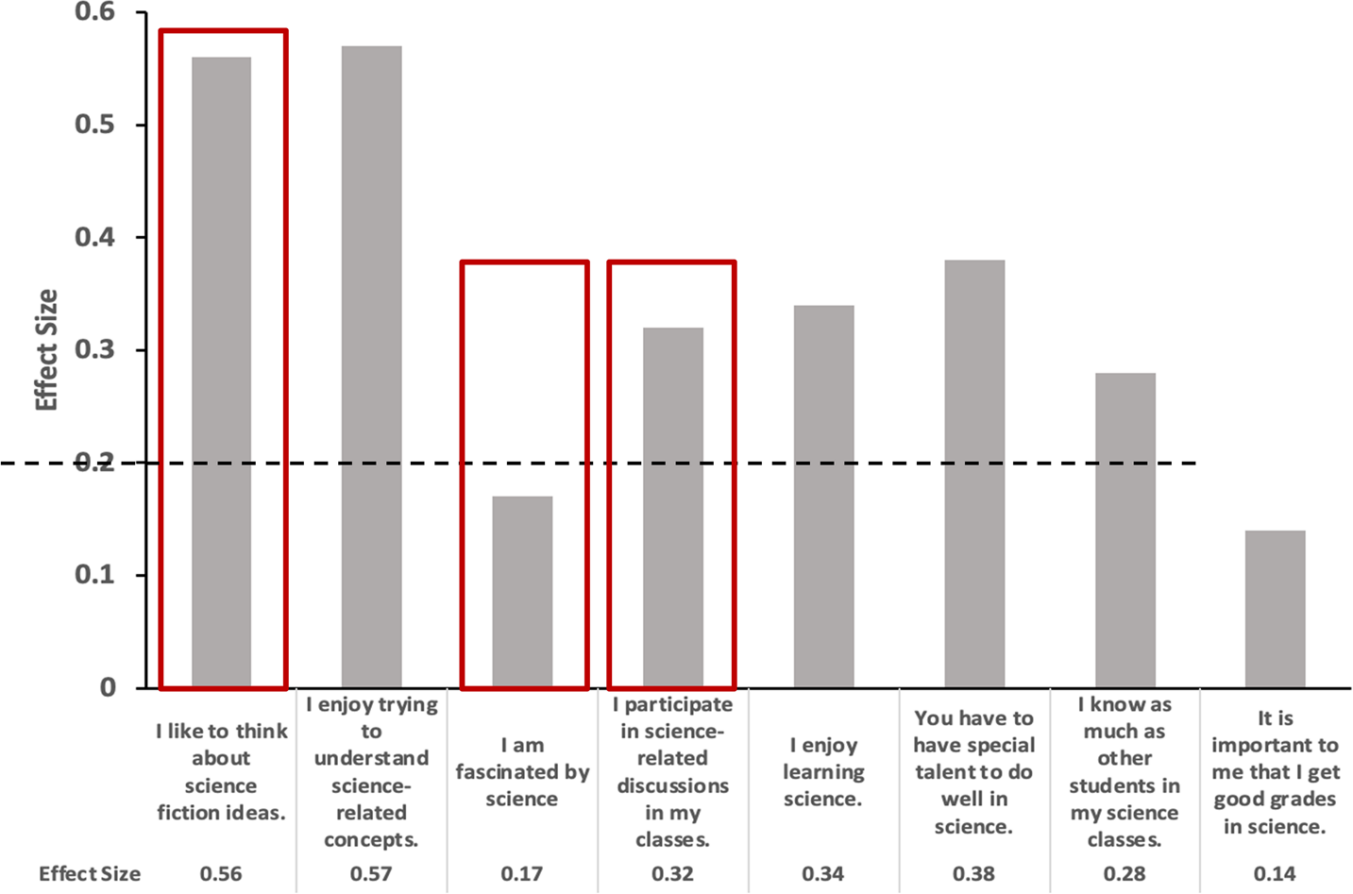
Effect sizes were calculated for all of the items on the survey. The data show the list of questions that were probed in order to ascertain an impact on the high school students’ perception of science. The dotted line is indicative of the threshold above which the effect sizes are statistically significant. The red boxes highlight items that had the largest effect sizes to the most impactful questions.

Students selected four-point Likert scale responses to eight statements about science and scientists. Integral weights were assigned for statistical analysis purposes with 1 = disagree strongly, 2 = disagree somewhat, 3 = agree somewhat, and 4 = agree strongly. The independent *t* test was used to test for pre/post mean differences at the 0.05 level of significance with the null hypothesis: difference = 0 and alternative hypothesis: difference < > 0. Effect sizes were then calculated for all the items on the survey. The statements of items, effect size, and thresholds of the effect size are displayed in Figure 3.

Overall, the effect sizes from the pre- and postsurveys exploring the impact of the laboratory activities on the high school students’ views of science and scientific relevance were indicative of being statistically significant. As Figure 3 shows, the interpretation of effect size used in this report is distributed as 0.8 (large), 0.5 (medium), and 0.2 (small). Medium effect sizes were measured for the statements “I enjoy trying to understand science-related concepts” and “I like to think about science fiction ideas” and were deemed extremely impactful. Other statements that also alluded to a statistically significant change (albeit a “small” effect size) in science perception are “I participate in science-related discussions in my classes” and “I enjoy learning science”. It is important to point out that the students were assessed after a short 6 weeks of interacting with the XULA students and performing acid–base experiments. It can only be assumed that with time and sustained experiential learning the impact would be more pronounced.

As such, written comments from the students were indicative of a better understanding of scientists and the work that scientists do. The students were asked to respond to the prompt: Think about what you know about scientists and describe what they do. The comments below summarize the sentiments of most of the responses:

*“Scientists perform research and conduct experiments to gain knowledge on a topic.”*

*“Scientists solve problems and help to make the world better and smarter.”*

“*An individual who studies a certain branch of science and experiments and tests different ideas relative to the branch of science they study.”*

The “*Is Science Me?*” survey was also instrumental in probing any impact on the career interests of the participating high school students. As Table 3 indicates, the survey gauged interest in ten different disciplines ranging from medical sciences to space science. Students recorded their interest (very interested, somewhat interested, not interested) both at the start of the school visits (before activities) and at the end of the six-week period.

**Table 3:** Students Recorded Their Interest (Very Interested, Somewhat Interested, Not Interested) in Ten Listed Science College Majors^a^

	Pre-Fall Interest			Post-Fall Interest		
College Major	Not	Somewhat	Very	Not	Somewhat	Very
Biology (Marine, Plant, Genetics, etc.)	73	23	4	41	36	23
Chemistry to Biochemistry	54	35	11	18	55	27
Physics	69	23	8	50	32	18
Environmental Science (Geology, Ecosystem Management)	65	23	12	36	36	27
Space Science (Astronomy)	61	11	27	36	36	27
Engineering	27	38	35	23	36	41
Medicine (Dentist, Medical Doctor, Physician’s Assistant)	58	31	11	29	43	29
Computer Science	38	31	31	27	46	27
Social Science	69	19	12	59	32	9
Psychology	69	15	15	36	46	18

a Frequency distributions of pre/post ratings for interest in college science majors are shown. These data were determined from “*Is Science Me?*” surveys administered before the initiation of the Fall 2022 classroom activities and after the acids–bases modules were completed at the end of the six-week visit.

The data show a marked difference with large to medium effect sizes (not shown) for Biology, Chemistry, Biochemistry, Environmental Science, Medicine, and Psychology. These preliminary findings are indicative of a successful impact on the career interests of the participating students with respect to the physical and biological sciences. Also of interest is the drastic decrease in the “not” category from the pre- to the postfall interest surveys.

### Pre- and Postdiscussion Surveys

**Figure 4:**
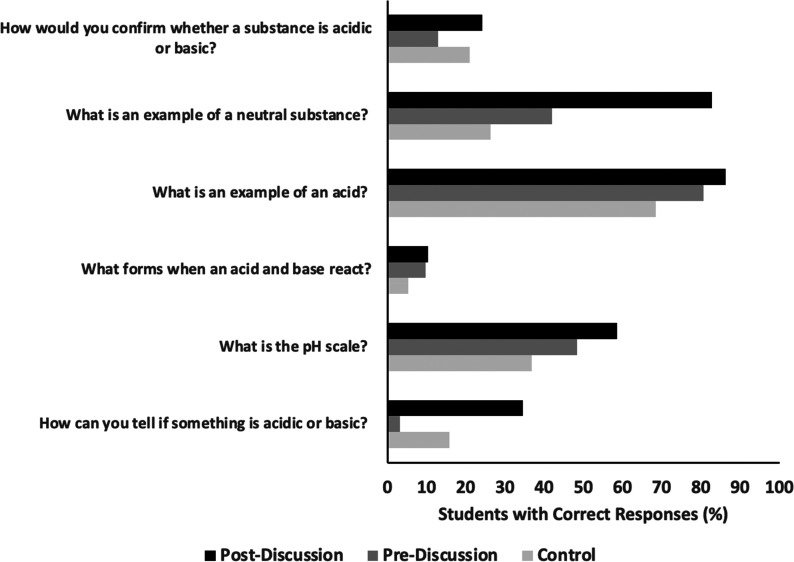
Post-test, pretest, and control classroom results (% of students answering correctly).

Summative assessments were provided via pre- and postdiscussion surveys. Both the pre- and postsurveys were identical questions exploring content knowledge about the broad topic area of acids and bases (specific questions shown in Figure 4).

On the first day of classroom visits, high school students were asked to fill in the prediscussion survey. The postdiscussion comprising identical questions was administered after the completion of all modules pertaining to the acid–base topic that we covered during the 6 weeks period.

We left space under each of these questions to allow students the freedom to choose whether to write or even draw the best answer possible. During data processing, one point was awarded for each correct response to a question, and hence, a maximum of six points could be earned on the prediscussion and six on the postdiscussion by each individual student. If multiple different responses or no responses were recorded on the question by the student, then it was assigned zero points. Also included was a comparison classroom within the same grade that did not receive any XULA-MOLE visits during the semester. The comparison group was given the postdiscussion survey at the end of the semester to account for any changes due to classroom discussions.

This *exploratory outcome evaluation* specifically determined which changes in student content knowledge can be attributed to participation in the XULA-MOLE project and whether students in the treatment group show mastery of the material in the inquiry-based modules with respect to the comparison group. The outcomes comparing the prediscussion, postdiscussion, and comparison classroom assessments clearly show that students in our treatment classroom answered noticeably more questions correctly on the postdiscussion, about acid–base content knowledge as seen in Figure 4. The percentages were calculated from the total number of students taking the survey for prediscussion (*n* = 31), postdiscussion (*n* = 29), and comparison (*n* = 19). However, because of the anonymous data collection, we could not correlate any movement for a specific high school student.

The data in Figure 4 are strongly indicative of an increase in acid–base content knowledge on the participating high school students.

### CER and Group Presentations

An auxiliary goal of our project was to prepare high school students to communicate their science understanding effectively and efficiently. Writing out a claim-evidence-reasoning (CER) discussion was an additional strategy to help students make connections between science concepts and laboratory activities by analyzing and interpreting their experimental results and applying them to a new problem. During the fifth week of the school visits, the high school students were provided with an article on dental erosion.^28^ The students were expected to correlate acid decay in teeth with acid–base reactions and experiments performed and discussed during prior weeks. As part of the CER profile, the high school students were required to summarize their engagement and learning from the previous 6 weeks and apply it to their discussion of tooth decay.

For the CER reflection, the high school students worked in groups to prepare posters or a video presentation depending on their comfort level. The videos and posters were independently graded by Xavier faculty, not involved in the XULA-MOLE project, using rubrics created by the project faculty. The presentations were assessed using a 5-point scale for content, accuracy, group participation, and effort, respectively. All grade distributions for the posters and presentations were above 3.8.

The posters and videos created by high school student groups were not all technically rigorous but are an independent indication of their efforts to communicate the learning outcomes of the project and served as an effective summative assessment of the project along with the postdiscussion questions.

### NGSS Outcomes

The learning outcomes/processes and experimental concepts are shown in Table 4 along with the NGSS^29^ (NGSS Acid–Bases) processes that this acid–base topic tried to reinforce. Included within each guiding question were a series of experiments that helped provide a foundation for an NGSS-aligned topic area. Each of these investigations asked the students to stop and consider one or more important concepts or issues connected to the leading question. This guided inquiry is predicted to support the student in developing critical thinking skills and practice with the State Common Core’s use of the CER rubric.^30^

**Table 4:** Design of Learning Objectives/Processes^24^ for “What Causes Degradation of New Orleans Cemeteries”

Learning Objectives	Processes for Students	Experimental Concepts
How do acids and bases form?	Observe	Acid–Base Classification
How can you tell if something is acidic or basic?	Hypothesize	Acid–Base Properties and Conductivity
Can you classify chemicals using known properties such as pH, conductivity, and litmus tests?	Predict	Models of acid base theory
How would you neutralize an acid or a base?	Create	Acid–Base neutralization
Can you use the Law of conservation of matter to determine the chemical composition of compounds and chemical reactions?	Infer	pH scale and litmus paper
**NGSS Standards: HS-PS1–2**: Construct and revise an explanation for the outcome of a simple chemical reaction.	Classify	
**HS-PS1–7**: Use mathematical representations to support the claim that atoms, and therefore mass, are conserved during a chemical reaction.	Measure	
**Disciplinary Core Ideas**: Asking questions, planning and carrying out investigations, analyzing and interpreting data, constructing explanations, engaging in argument from evidence (CER).	Experiment	

By the end of the semester, students were able to communicate how acids and bases form; understand the pH scale and use a calculator to compute pH from hydrogen ion concentrations and vice versa; differentiate between acids and bases depending on their properties; classify a substance as acidic, basic, or neutral based on pH or hydrogen ion concentration and conductivity and use these lessons to explore the leading question of the degradation of New Orleans cemeteries as required by the textbook.

### Benefits to XULA-MOLE Students

The data also indicate the impact of this project and the related activities on XULA-MOLE near-peer mentor students. As shown in Table 5, the XULA-MOLE undergraduate students reported a change in perceptions of their own teaching abilities and skills. The students felt more ready for science communication and teaching, indicating positive impacts on their own career aspirations (a more detailed version of Table 5 is available in the Supporting Information) As such, a secondary outcome of this outreach project was an increased understanding of science concepts for the XULA-MOLE undergraduate students.

**Table 5:** Quotes^a^ from XULA Volunteers Regarding Their Own Experiences and of Other Students

Self-Reflection of Project by the XULA-MOLE Students	Reflections on High School Students’ Experience by the XULA-MOLE Students
“Personally I found the experience enjoyable as I was able to form connections with the students and see their excitement when they were able to recall information from previous experiments and apply the information to new labs.”	“The students in my classroom enjoyed the experiments and felt like they were learning and understanding tough concepts. The students also indicated that they enjoyed our visits to their schools. They enjoyed them so much that they were upset that we would be teaching a new class of students in the next semester.”
“I feel that I am making a difference even if it’s a small one. It’s good that we are doing what we can to increase STEM opportunities for students that look like me.”	“Many of the kids were excited about STEM. They loved seeing the reactions take place and when we would ask them preliminary questions they were very creative about their answers.”
“I loved volunteering at the high school. I loved building a mentor relationship with the students. Them asking me advice in relation to their future was the highlight of my experience”	“The wet labs themselves made the high schoolers feel like scientists especially when they could observe a physical change (like a color change or gaseous formation). Whenever I asked them to pick a group leader, they consolidated with a lot of pride because who ever was the group leader I called them “head scientist”. They were always excited to see us, and often asked really good questions.”
“I looked forward to very outreach day because i was just excited to see them excited and to be a part of their excitement. It reminded me of myself at my earliest encounters with Chemistry. I felt like I was igniting a flame in them that was just hungry for more experiments, which was an incredibly rewarding experience.”	“A lot of students hadn’t seen or met a lot of young black people in stem so it shocked a lot of them especially if one day I went in my scrubs. It really made a lot of them think and lean on each other more when there was a difficult topic. Most of them didn’t realize the wide variety of topics and fields that can be experienced through STEM so they seemed more open to learning after speaking with us.”

a Quotes here are used verbatim and not modified for comprehension and/or grammar.

As mentioned earlier, an important outcome of the project included the near-peer mentoring relationship that resulted between the XULA students and the middle/high school students from the school visits. Peer-to-peer mentorship:

i. Transformed the XULA students from being role models to trained mentors.
ii. Expanded the pool of high school participants who were impacted by the near-peer discussion sessions.

Both high school students and XULA students appreciated this outcome of the mobile service-learning initiative. Although peer mentoring was not quantitatively assessed, the XULA student volunteers and Team Leads reported that during this opportunity, the high school students had many nonchemistry questions to discuss with the XULA students with respect to applying to college, financial aid, and career aspirations. Volunteers expressed a sense of fulfillment in interacting with and mentoring younger students. The XULA near-peer mentors thoroughly enjoyed the mentorship role as gauged by qualitative surveys and reflections shown in Table 5 (and in the Supporting Information). They reported this as an ideal mechanism to give back to the community that is also aligned with the mission of XULA.

### Challenges

One of the first challenges besides the pandemic and the hurricane, which delayed the initiation of this project, was the high turnover of science teachers at the participating schools. Despite our efforts to contact over 15 local schools, only four responded. We are addressing this challenge by building more sustainable means of communication with the teachers, which are independent of their school profiles.

Enlisting XULA student volunteers for the time slots chosen by teachers, cancellation of visits due to unannounced school events, and personal emergencies of the teachers also delayed our visit weeks. Student-led development of experiments and wait times for chemicals and materials to arrive beforehand were also a challenge, especially since we wanted XULA volunteers to take a leadership role in the design of the experiments.

### Strengths and Accomplishments

There were several advantages of conducting this mobile laboratory project, including the ability to provide resources to four participating high schools.

*Improving access to STEM education*: By bringing experimental materials to the students, opportunities for science enrichment like field visits to science museums and academic institutions are replicated. The mobile laboratory project enabled students to engage in scientific enrichment activities and interactions with STEM-trained undergraduate students during their regular class time.

*Limiting the schools’ burden*: By offering this program free of cost to the schools, there was minimal budgetary strain on the schools. Since the classes were held during the routinely scheduled class time, there was no interference with any other activities or events planned by the schools and worked to reinforce the topics being discussed in the classroom. Moreover, no time was wasted transporting the students from their schools to off-site locations.

This program allowed students to access and experience authentic laboratory hands-on training that school teachers may not have had the resources or necessary bandwidth to provide. During this project period, the teachers received resources in terms of curriculum-related modules and experiments that they could use and implement in their future classes.

XULA-MOLE students and high school students developed a strong mentor-and-mentee relationship through repeated interactions over the six-week project. XULA-MOLE students would refer to the school classrooms as “*my class*” while the teachers would text us and say “*students are asking and waiting for their XULA friends*”.

Leadership and confidence levels of XULA-MOLE students were bolstered, as reported by them in their self-reflections (Table 5). As peer mentors, they assisted the high school students each week with the assigned experiments and provided a safe discussion space. The hands-on training the XULA students received reinforced their training as future leaders in their community.

### Future

For our project moving forward, we will try to address the challenges posed especially with respect to reaching additional classrooms and adapting to teacher turn-around. We will also carry out statistical evaluations to correlate pre- and postdiscussion questions to additional parameters such as gender, race/ethnicity, socioeconomic advantages, etc.

## Conclusions

A student’s foundation in science is constructed from a diverse set of formal and informal experiences. The discussed XULA-MOLE project offers a framework for implementing “hands-on” STEM principles within participating high schools with underserved majority populations while providing numerous opportunities for informal STEM mentorship and training via near-peer interactions. By working closely with XULA-MOLE students, the high school students were able to impact their reflections on how a career in STEM may be accessed and how different types of opportunities may be available to them in the future.

The described initiative was unique because it not only increased exposure to STEM fields but also fostered increased interest and appreciation for future careers in STEM among the participating student population. The impact of this project is evident in student understanding of scientific concepts, their engagement with science, and their views on the STEM fields, potentially leading to an increase in diversity of action and thought in their future careers.

There is great potential for replicating this approach in other outreach programs or modifying these activities to suit the needs of the participating communities and institutions.

## Supporting Information Available

Acid–Bases Week 4 Datasheet (PDF)
Acid–Bases Week 4 Potential Lecture (PDF)
Supporting figure and table (PDF)
