# Supplementary material for: Impacting Career Choices of Historically Underserved Secondary Students by Designing Near-Peer Directed Acid–Base Thematic Laboratory Activities to Enhance STEM Interest
Source: J Chem Educ. 2023 Aug 21;100(9):3434–44. doi: 10.1021/acs.jchemed.3c00434 (PMC10501114; doi:10.1021/acs.jchemed.3c00434)
Supplement: Supplementary file 1 — ed3c00434_si_001.pdf [file ed3c00434_si_001.pdf]

Supporting Information

Impacting Career Choices of Historically Underserved Secondary Students by Designing Near-Peer Directed Acid-Base Thematic Laboratory Activities to Enhance STEM Interest

Abha Verma* and Mehnaaz F. Ali

Department of Chemistry, Xavier University of Louisiana, New Orleans, Louisiana-70125, USA.

*Corresponding Author Email: averma1@xula.edu

LINKS TO ORIGINAL ACID-BASE MODULE-4 FILES

https://drive.google.com/drive/u/1/folders/1837vTNNMtEBeTIC0PVQXTFkI1gLtd7HA

XULA_MOLE_ACID-BASE - Google Drive

LINKS TO XULA-MOLE WEBSITE

https://www.xula.edu/mole/index.html

XULA MOLE | Xavier University of Louisiana

XULA MOLE_Week-4 _ Effect of Acid-rain on Cemeteries experiment Data Sheet

Key for observations:<br><br>1. Color Change (cc)<br>2. Precipitation/solid formation (ppt)<br>3. Effervescence / Bubbles rising (Gas)<br>4. Other?	**Hypothesis:** Which of the stones will react the fastest? Why?

Place your reaction vials here ⇨						
**1 mL =**<br><br>**18-22 drops**	**Acid 1**<br><br>**0.5 M HNO_3**	**Acid 2**<br><br>**1 M HNO_3**	**Acid 3**<br><br>**3 M HNO_3**	**Acid 4**<br><br>**0.5 M H_2SO_4**	**Acid 5**<br><br>**1 M H_2SO_4**	**Acid 6**<br><br>**3 M H_2SO_4**
**Observation with**<br><br>_______________						
**Time of Reaction with**<br><br>_______________						
**pH of reaction solution with**<br><br>_______________						

**Observation/ Time/pH with Granite/$CaCO_3$**						

20
