# Supplementary material for: Impacting Career Choices of Historically Underserved Secondary Students by Designing Near-Peer Directed Acid–Base Thematic Laboratory Activities to Enhance STEM Interest
Source: J Chem Educ. 2023 Aug 21;100(9):3434–44. doi: 10.1021/acs.jchemed.3c00434 (PMC10501114; doi:10.1021/acs.jchemed.3c00434)
Supplement: Supplementary file 2 — ed3c00434_si_002.pdf [file ed3c00434_si_002.pdf]

Supporting Information

Impacting Career Choices of Historically Underserved Secondary Students by Designing Near-Peer Directed Acid-Base Thematic Laboratory Activities to Enhance STEM Interest

Abha Verma* and Mehnaaz F. Ali

Department of Chemistry, Xavier University of Louisiana, New Orleans, Louisiana-70125, USA.

*Corresponding Author Email: averma1@xula.edu

LINKS TO ORIGINAL ACID-BASE MODULE-4 FILES

https://drive.google.com/drive/u/1/folders/1837vTNNMtEBeTIC0PVQXTFkI1gLtd7HA

XULA_MOLE_ACID-BASE - Google Drive

LINKS TO XULA-MOLE WEBSITE

https://www.xula.edu/mole/index.html

XULA MOLE | Xavier University of Louisiana

The potential lecture slides presented here were prepared by XULA-MOLE undergraduate student Team Leads (XUSTL) during Fall of 2022.

Acid-Base_Week-4

Lecture points to be covered:

1. Review Acid-Base
2. Reactions of Acid-Base
3. Neutralization reactions
4. Mention about concentrations, units of concentration (Molarity, etc)
5. Effect of Concentrations/Dilutions on pH
6. How Acid Rain forms? (Pollution, etc.)
7. Effect of acid-rain on land, crops and especially on Cemetery stones

Materials needed for each group of 4-5 students:

1. 4 scintillation vials
2. 2 acid dropper bottles (0.5 M HNO_3, 1 M HNO_3 and 3 M HNO_3 OR 0.5 M H_2SO_4, 1 M H_2SO_4 and 3 M H_2SO_4)
3. pH paper
4. Timer
5. Watch glass
6. Stirring rod
7. Stone chips: Granite, Quartz, Marble or Chalk (each group gets only 2 types of stones. Choose between Granite or Chalk ($CaCO_3$) AND Quartz or Marble)
8. Datasheet
9. DI water bottle
10. Kim Wipe
11. Googles
12. Waste Bottle

Potential Lecture Slides:

	Composition	Picture
**Marble**	38–42% Lime (CaO), 20–25% Silica (SiO2), 2–4% Alumina (Al2O3), 1.5–2.5% oxides (NaO and MgO), and 30–32% (MgCO3 and others)	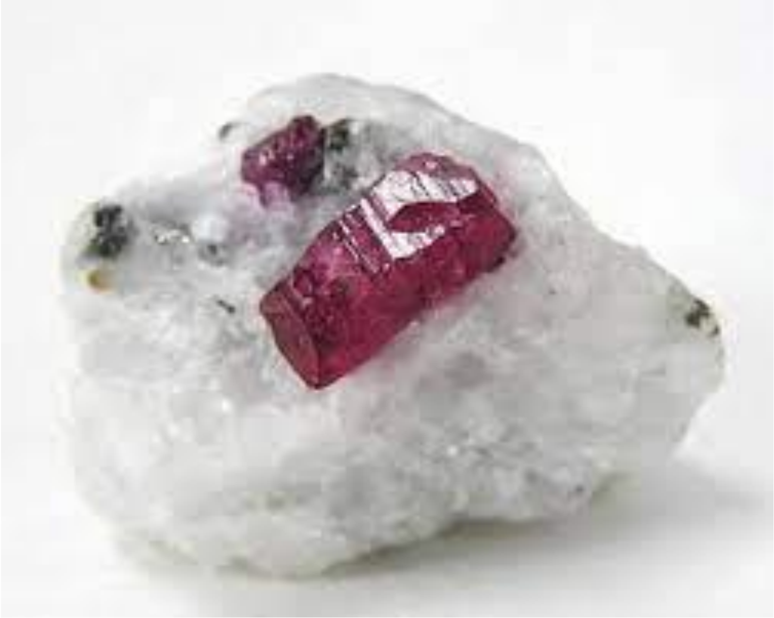

**Granite**	70-77% silica, 11-13% alumina, 3-5% potassium oxide, 3-5% soda, 1% lime, 2-3% total iron, and less than 1% magnesia and titania	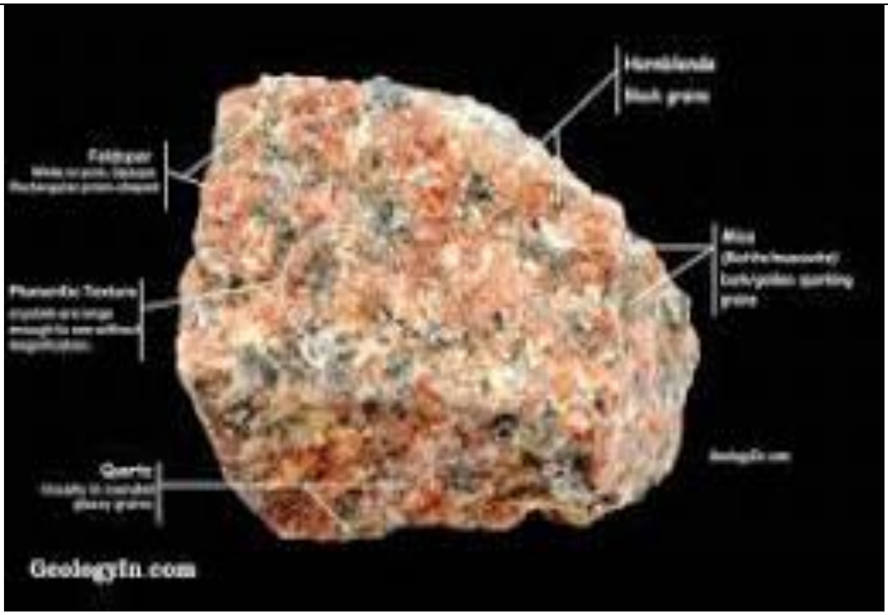
**Quartz**	Quartz is a hard, crystalline mineral composed of silica (silicon dioxide). The atoms are linked in a continuous framework of SiO_4 silicon-oxygen tetrahedra, with each oxygen being shared between two tetrahedra, giving an overall chemical formula of SiO_2	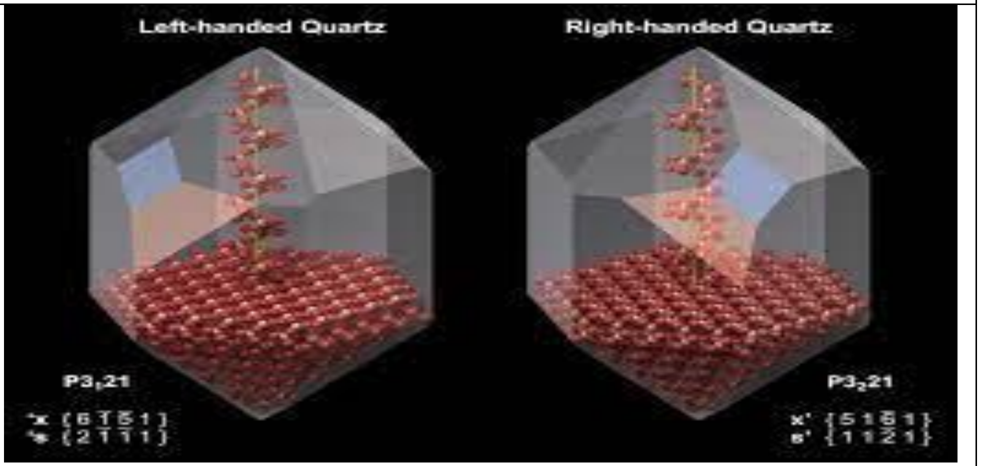
**Chalk**	CaCO3 (sedimentary rock)	

**Quartzite**	predominantly quartz (quartz content in the rock reaches 88-98%), with iron hydroxides (2-3%), silicon and chalcedony (4-5%). The stone also contains impurities of mica, talc, feldspar, and other minerals.	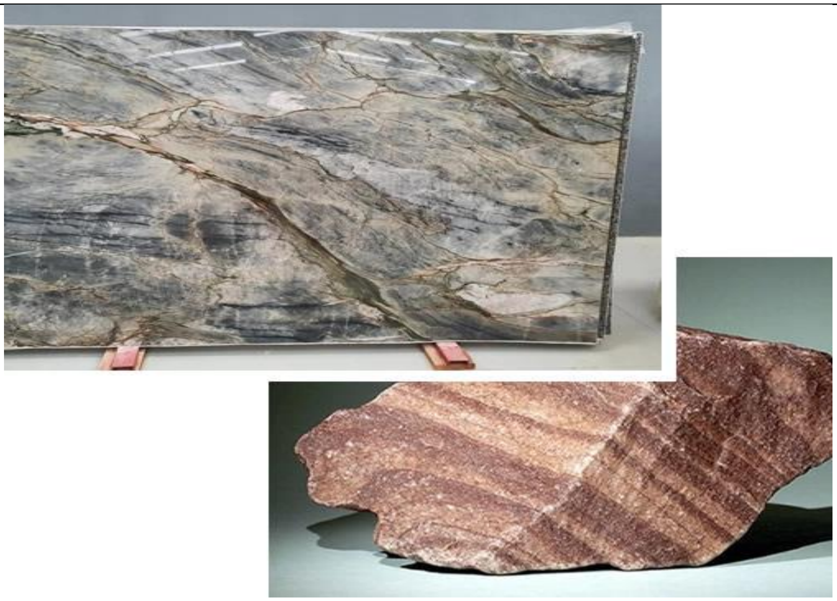

References:

1. https://www.veneziasurfaces.com/about/articles/141-quartzite-properties-composition-mining-quartzite-in-the-interior#:~:text=The%20Composition%20of%20Quartzite.&text=The%20mineral%20composition%20of%20quartzite,%2C%20feldspar%2C%20and%20other%20minerals.
2. http://www.actforlibraries.org/the-chemical-composition-of-marble/
3. https://www.irocks.com/minerals/specimen/16942
4. https://nature.berkeley.edu/classes/eps2/wisc/granite.html
5. https://www.geologyin.com/2015/11/what-is-granite-and-how-is-it-formed.html
6. https://mediakron.bc.edu/environmentalissues/topics/quartz-crystal-rock-crystal
7. https://www.mindat.org/mesg-562212.html
