# Supplementary material for: Impacting Career Choices of Historically Underserved Secondary Students by Designing Near-Peer Directed Acid–Base Thematic Laboratory Activities to Enhance STEM Interest
Source: J Chem Educ. 2023 Aug 21;100(9):3434–44. doi: 10.1021/acs.jchemed.3c00434 (PMC10501114; doi:10.1021/acs.jchemed.3c00434)
Supplement: Supplementary file 3 — ed3c00434_si_003.pdf [file ed3c00434_si_003.pdf]

Supporting Information

Impacting Career Choices of Historically Underserved Secondary Students by Designing Near-Peer Directed Acid-Base Thematic Laboratory Activities to Enhance STEM Interest

Abha Verma* and Mehnaaz F. Ali

Department of Chemistry, Xavier University of Louisiana, New Orleans, Louisiana-70125, USA.

*Corresponding Author Email: averma1@xula.edu

LINKS TO ACID-BASE MODULE-4 FILES

https://drive.google.com/drive/u/1/folders/1837vTNNMtEBeTIC0PVQXTFkI1gLtd7HA

XULA_MOLE_ACID-BASE - Google Drive

LINKS TO XULA-MOLE WEBSITE

https://www.xula.edu/mole/index.html

XULA MOLE | Xavier University of Louisiana

For our program, the high school students worked in groups to prepare posters as indicated in Figure S1, or a video presentation, depending on their comfort level. The videos and posters were independently graded by Xavier faculty, not involved in the XULA-MOLE project, using rubrics that were created by project faculty. The presentations were assessed on a 5-point scale and assessed on content, accuracy, group participation, and effort. All grade distributions for the posters and presentations were above 3.8.

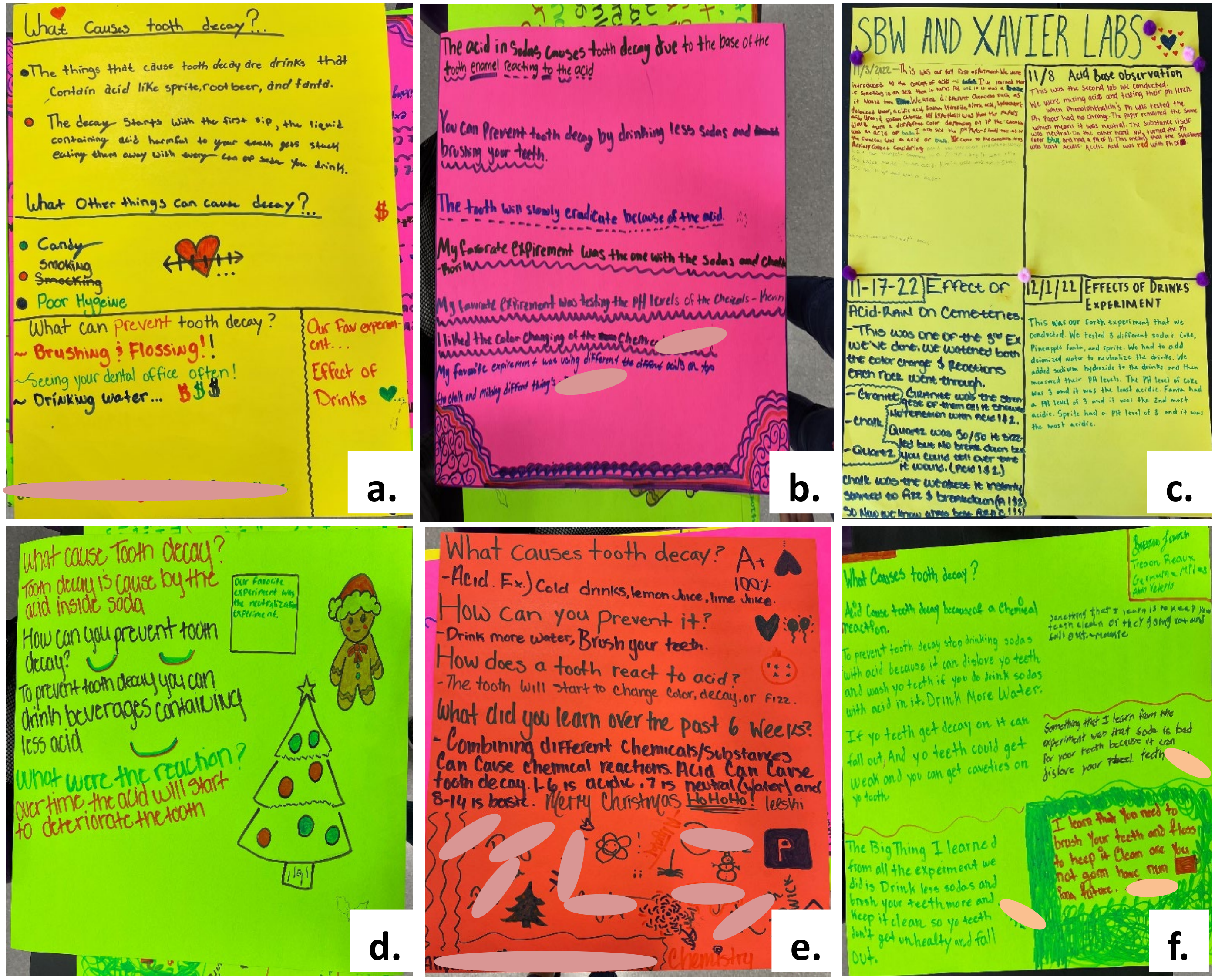

Figure S1. Discussion boards and posters (a-f) prepared by high school students at the end of semester by different student groups. The names of the students are concealed, since we only have permissions for pictures and the student names to be anonymous.

The data also indicates the impact of this program and the related activities on XULA-MOLE volunteer students. As shown in Table 5 (detailed version from the manuscript file), the XULA-MOLE students reported a change in perceptions of their own teaching abilities and skills. The XULA-MOLE students felt more ready for science communication and teaching, indicating positive impacts on their own career aspirations. As such a secondary outcome of this outreach project was an increased understanding of science concepts for the XULA-MOLE students.

Table 5. Quotes* from XULA volunteers regarding their own experiences and of other students'

**Self-reflection of project by XULA-MOLE students**	**Reflections on high school students experience by the XULA-MOLE students**
"Personally I found the experience enjoyable as I was able to form connections with the students and see their excitement when they were able to recall information from previous experiments and apply the information to new labs."<br><br>"I absolutely love using my chemistry laboratory experience to help educate high schoolers and expose them to what isn't available to them. I love supporting students in their interest in STEM."<br><br>"I feel that I am making a difference even if it's a small one. It's good that we are doing what we can to increase STEM opportunities for students that look like me."<br><br>"I loved volunteering at the high school. I loved building a mentor relationship with the students. Them asking me advice in relation to their future was the highlight of my experience"<br><br>"Volunteering with this program made me feel like I've made a difference. In the beginning you could tell the students were learning the information but didn't truly understand why. So, swing that change over time made me feel good and accomplished with our goals."<br><br>"It made me feel happy and like I really made a difference. I remember being that kid in science classes wanting to learn more and being excited about STEM, so i feel like I can relate to them a lot"<br><br>"I looked forward to very outreach day because i was just excited to see them excited and to be a part of their excitement. It reminded me of myself at my earliest encounters with Chemistry. I felt like I was igniting a flame in them that was just hungry for more experiments, which was an incredibly rewarding experience."<br><br>"I enjoyed working with highschoolers and i'm glad I got to participate in this program and make a difference in students lives. I enjoyed them and their	"Students were very engaged in experiments where they could manipulate the outcome on their own or witness physical changes. For example, one student said he felt like a scientist upon testing the effect of acid on a stone, which is the noble purpose of this project."<br><br>"The students in my classroom enjoyed the experiments and felt like they were learning and understanding tough concepts. The students also indicated that they enjoyed our visits to their schools. They enjoyed them so much that they were upset that we would be teaching a new class of students in the next semester."<br><br>"The high schoolers were very excited about the reactions and appreciative of receiving a visual representation of what they were learning in chemistry."<br><br>"Many of the kids were excited about STEM. They loved seeing the reactions take place and when we would ask them preliminary questions they were very creative about their answers."<br><br>"Many of the kids were excited about STEM. They loved seeing the reactions take place and when we would ask them preliminary questions they were very creative about their answers."<br><br>"I believe the activities increased the students interest in science. It made the things they were learning on paper into real life scenarios. I definitely think it made them feel good about themselves and peaked interest into the science field. Also, you could see the increase in confidence as the weeks went by when it came to handling the materials."<br><br>"The wet labs themselves made the high schoolers feel like scientists especially when they could observe a physical change (like a color change or gaseous formation). Whenever I asked them to pick a group leader, they consolidated with a lot of pride because who ever was the group leader I called them "head scientist". They were always excited to see us, and often asked really good questions."<br><br>"A lot of students hadn't seen or met a lot of young black people in stem so it shocked a lot of them especially if one day I went in my scrubs. It really made a lot of them think and lean on each other more when there was a difficult topic. Most of them didn't realize the wide variety of topics and fields that can be experienced through

curiosity about the new things they were learning. I felt like a huge role model to the students.”	STEM so they seemed more open to learning after speaking with us.”<br><br>“The high-schoolers enjoyed participating in the activities as they expressed that they have never participated in such experience. They enjoyed being about to apply what they were learning to the experiments. They also enjoyed the mentorship as they were able to learn more about college and from a science major viewpoint.”

**Quotes here are used verbatim and not modified for comprehension and/or grammar.*
